# WWP2 is required for normal cell cycle progression

**DOI:** 10.18632/genesandcancer.83

**Published:** 2015-09

**Authors:** Byeong Hyeok Choi, Xun Che, Changyan Chen, Luo Lu, Wei Dai

**Affiliations:** ^1^ Department of Environmental Medicine, New York University Langone Medical Center, Tuxedo Park, NY, USA; ^2^ Center for Drug Discovery, Northeastern University, Boston, MA, USA; ^3^ Division of Molecular Medicine, Department of Medicine, David Geffen School of Medicine, University of California Los Angeles, Torrance, CA, USA

**Keywords:** WWP2, Cell Cycle, Cyclin, Mitosis, Ubiquitin E3 ligase, Akt

## Abstract

WWP2 is a ubiquitin E3 ligase belonging to the Nedd4-like family. Given that WWP2 target proteins including PTEN that are crucial for regulating cell proliferation or suppressing tumorigenesis, we have asked whether WWP2 plays a role in controlling cell cycle progression. Here we report that WWP2 is necessary for normal cell cycle progression as its silencing significantly reduces the cell proliferation rate. We have identified that an isoform of WWP2 (WWP2-V4) is highly expressed in the M phase of the cell cycle. Silencing of WWP2 accelerates the turnover of cyclin E, which is accompanied by increased levels of phospho-histone H3 (p-H3) and cyclin B. Moreover, silencing of WWP2 results in compromised phosphorylation of Akt^S473^, a residue whose phosphorylation is tightly associated with the activation of the kinase. Combined, these results strongly suggest that WWP2 is an important component in regulating the Akt signaling cascade, as well as cell cycle progression.

## INTRODUCTION

Nedd4-like proteins are a protein family of ubiquitin E3 ligases which share several similar structures including an N-terminal C2 domain, WW (double tryptophan) domains, and a C-terminal HECT domain. Nedd4-like proteins frequently target proteins that are involved in regulating cell cycle and apoptosis. WWP2 (WW domain containing E3 Ub-protein ligase 2) is a member of Nedd4-like family, which was first identified in 1988 and shown to regulate protein-membrane interaction [1, 2]. As an ubiquitin E3 ligase, WWP2 targets PTEN and mediates its proteolytic degradation [3]. WWP2 also targets Oct-4, a transcription factor that plays a crucial role in the maintenance of pluripotency of stem cells. Extensive studies have also identified several other important targets of WWP2 E3 ligase that include ion channels and proteins involved in the immune responses [4-6]. Independent of its ubiquitination function, WWP2 is capable of binding Sox9, regulating its transcriptional activity by mediating its interaction with Med25 in palatogenesis [7].

WWP2 plays an important role in tumorigenesis as it directly targets oncogene products and/or tumor suppressors. For example, WWP2 polyubiquitinates PTEN, a major tumor suppressor, leading to the degradation of PTEN and enhanced Akt signaling pathway [3, 8]. WWP2 is also involved in epithelial-mesenchymal transition, as well as cell cycle arrest, by targeting Smads proteins in the TGF-β signaling pathway. Moreover, WWP2 isoforms exhibit tissue-specific expression patterns and have differential preferences with Smad proteins [9]. It has been suggested that WWP2 isoforms can be potentially used as prognostic markers, as well as therapeutic targets for certain malignancies [10]. In this study we report that WWP2 plays a role in cell cycle regulation as its silencing significantly reduces the cell proliferation rate. An isoform of WWP2 (WWP2-V4) was specifically expressed in M phase of the cell cycle. Silencing of WWP2 accelerated turnover of cyclin E, which was accompanied by increased levels of phospho-histone H3 (p-H3) and cyclin B. Moreover, silencing of WWP2 led to reduced activities of the Akt signaling pathway.

## RESULTS

### WWP2 regulates cell proliferation

**Figure 1:**
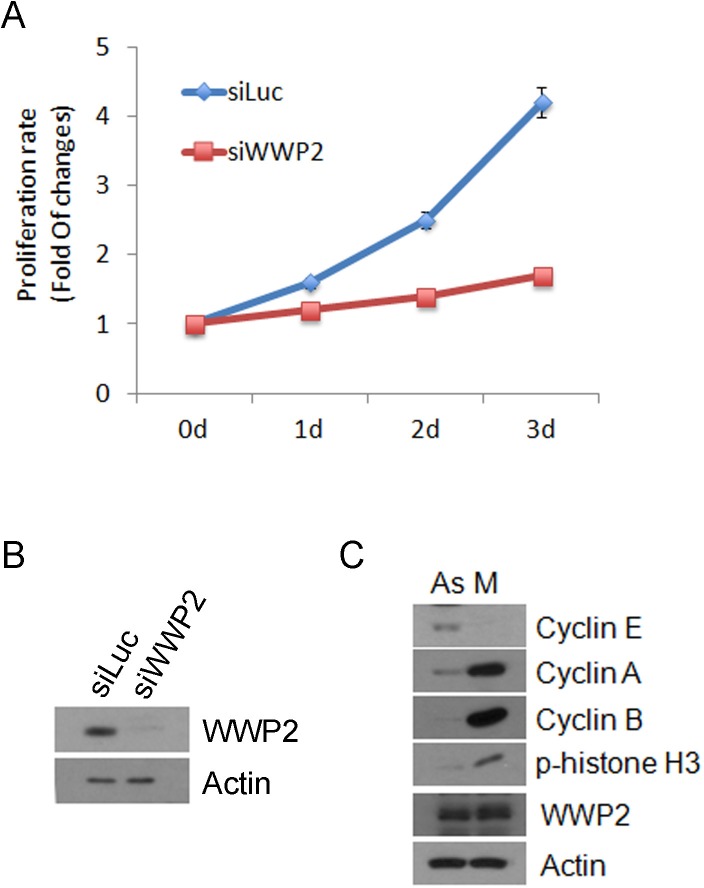
Knock-down of WWP2 inhibits cell proliferation **(A)** HeLa cells were transfected for 24 h with WWP2 siRNAs (siWWP2) or luciferase siRNA (siLuc) and cell proliferation were measured by CCK8 assay kit. Relative cell growth rates expressed as folds of change in the cell number were plotted. Averages of three experiments and their standard deviations (in brackets) are shown. (P<0.05). **(B)** HeLa cells were treated with (M) or without nocodazole (As) for 14 h. Cells were collected by and equal amounts of cell lysates, were prepared and blotted for cyclin E, cyclin A, cyclin B, phospho-histone H3, and actin.

To investigate whether WWP2 affect cell proliferation, HeLa cells were transfected with WWP2 siRNA or luciferase siRNA as control. Twenty-four h after transfection, the cell proliferation rate was measured. We observed that WWP2 knock-down significantly reduced HeLa cell proliferation (Figure 1A). Western blot analysis revealed that WWP2 was expressed in both asynchronized and mitotic (nocodazole-treated) cells (Figure 1B). There was no significant change in total WWP2 protein levels between the asynchronized and mitotic cell population. Analysis of cell cycle markers confirmed that mitotic arrest was efficient. Combined, these results indicate that WWP2 is involved in regulating cell proliferation.

### WWP2 isoform expression is increased during cell cycle progression

**Figure 2:**
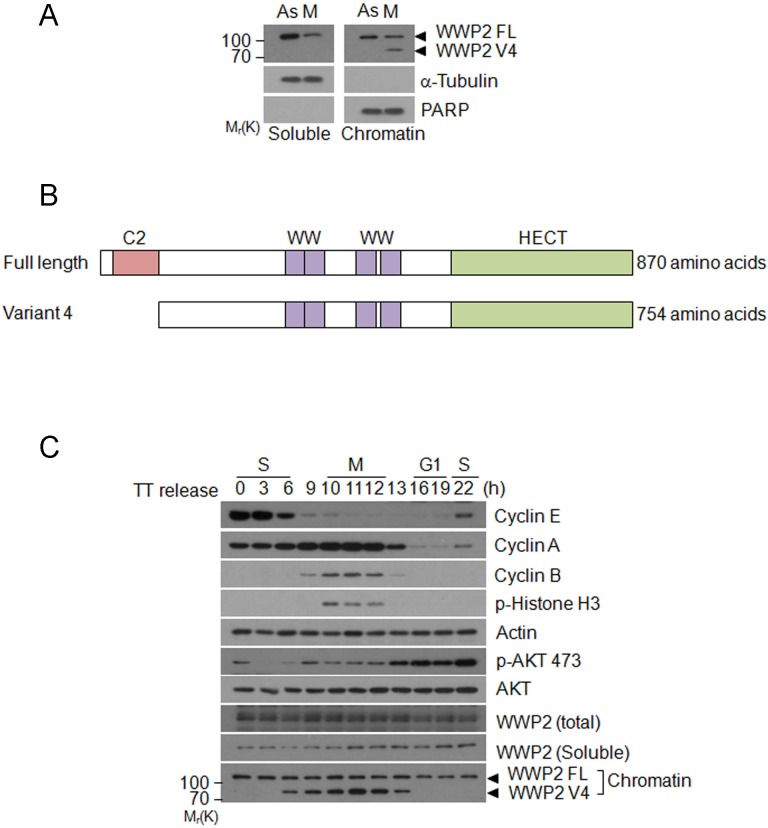
Expression of WWP2 transcription variant during the cell cycle **(A)** HeLa cells were treated with (M) or without nocodazole (As) for 14 h. Cells were then collected for obtaining the nuclear/chromatin fraction. Equal amounts of lysates from both groups were blotted for WWP2, PARP, and α-tubulin. **(B)** HeLa cells synchronized at the G1/S junction by the double-thymidine (TT) block were released into the cell cycle. At various times of release, cells were collected and fractionated into cytoplasmic/soluble and chromatin-bound fractions. Equal amounts of lysates from both fractions were blotted for cyclin E, cyclin A, cyclin B, phospho-H3, β-actin, phospho-Akt^S473^, total Akt, and WWP2. **(C)** Schematic presentations of full length human WWP2 and WWP2 transcription variant 4. C2 stands for C2-domain. WW stands for WW domain. HECT stands for HECT E3 ligase domain.

Given that WWP2 is a nuclear protein regulating polyubiquitination of Notch3 [11], we speculated that WWP2 protein might be localized to and have a role in the nucleous. To test this possibility, HeLa cells were treated with or without nocodazole. Nuclear (chromatin) fraction was isolated and blotted with the antibody to WWP2. We observed that WWP2 protein levels in the nucleus exhibited no significant changes between interphase and mitotic cells (Figure 2A). However, a new WWP2-specific band about 85 kDa, a spliced form named WWP2 V4 (Figure 2C), was detected during mitosis. Blotting with antibodies to PARP (primarily chromatin) and α-tubulin (primarily soluble) indicated that cellular fractionation was efficient.

There are six WWP2 isoforms that are derived from the same gene locus based on database search. Among these WWP2 isoforms is transcription variant 4, which is deleted in N-terminal C2 domain sequence that regulates the membrane association (Figure 2B). This isoform has 754 amino acids with a predicted molecular weight about 85 kDa. Therefore, based on the observed molecular weight, it is highly likely that the 85 kDa band is transcription variant 4 of WWP2. Further supporting it, WWP2 siRNA was capable of down-regulating this isoform.

To further confirm this finding, we synchronized HeLa cells at the G1/S junction by double thymidine block and then released them into the cell cycle. At various times of release, cells were collected and fractionated into soluble (cytoplasmic) and nuclear (chromatin) fractions. WWP2 protein levels in both soluble and chromatin fractions of various cell cycle stages were examined. WWP2 neither in the soluble (cytoplasmic) fraction nor in the chromatin fraction was significantly changed. However, in the chromatin fraction, WWP2 V4 started to appear during late S phase, peaking at mitosis (Figure 2B). This isoform disappeared roughly at the mitotic exit. Combined, these results indicate that WWP2 isoform may play a crucial role in mitotic entry, as well as mitotic progression.

### Down-regulation of WWP2 enhance M-phase arrests

**Figure 3:**
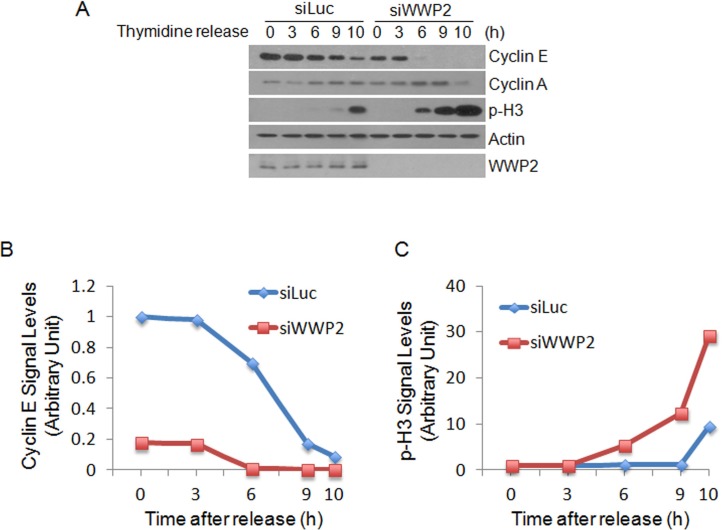
Knock-down of WWP2 arrests cells in M phase **(A)** HeLa cells transfected with WWP2 siRNAs (siWWP2) or luciferase siRNA (siLuc) were synchronized with thymidine block for 18 h. Cells were then released into the cell cycle by culturing in fresh medium. At various times post release, cells were collected and equal amounts of lysates were blotted with antibodies to cyclin E, cyclin A, phospho-H3, WWP2, and β-actin. **(B, C)** Cyclin E and phospho-H3 signals in cells transfected with siWWP2 or siLuc, as shown in panel A, were quantified and the summarized data were plotted.

To determine whether WWP2 affects cell cycle regulation, HeLa cells transfected with WWP2 siRNA or luciferase siRNA as control were synchronized with double-thymidine. These cells were then released into the cell cycle. At various times post release, cells were lysed and equal amounts of cell lysates were blotted with cyclin E and cyclin A. Transfection of WWP2 siRNA significantly decreased cyclin E. The half-life of cyclin E was decreased by at least 4.5 h in cells transfected with WWP2 siRNA as compared with cells with control siRNA (Figure 3A and 3B). Moreover, down-regulation of WWP2 significantly enhanced mitotic phosphorylation of histone H3 (p-H3) (Figure 3C). Taken together, these observations suggest that WWP2 plays a role in the regulation of transition of S phase into mitosis, as well as mitotic progression.

### WWP2 regulates mitosis progression

**Figure 4:**
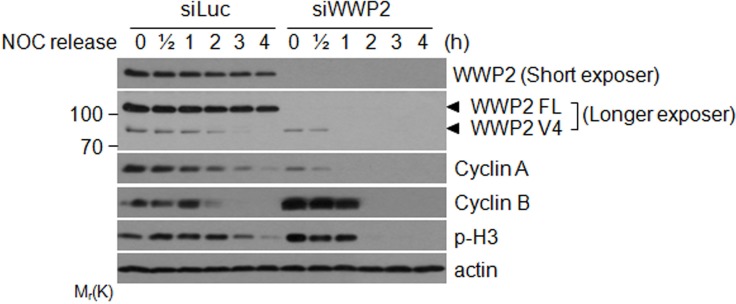
WWP2 is important for regulating mitotic progression HeLa cells transfected with WWP2 siRNAs (siWWP2) or luciferase siRNA (siLuc) for one day were treated with nocodazole for 14 h. Mitotic cells collected by shake-off were released into the cell cycle. At various times post release, cells were collected and fractionated into cytoplasmic/soluble and chromatin-bound fractions. Equal amounts of lysates from both fractions were blotted for WWP2, cyclin A, cyclin B, phospho-H3, and β-actin.

To further understand the role of WWP2 in regulating mitosis during cell cycle progression, HeLa cells transfected with WWP2 or luciferase siRNA were arrested at mitosis by nocodazole treatment and then released into the cell cycle. At various times post release, cells were lysed and equal amounts of cell lysates were blotted for cyclin B and p-H3. Silencing of WWP2 significantly increased levels of cyclin B and p-H3 but decreased cyclin A level in mitotic cells (Figure 4). Intriguingly, cyclin B and p-H3 signals were abruptly disappeared roughly 1 h after mitotic release, strongly suggesting that absence of WWP2 promotes mitotic exit. Silencing of both full length and spliced V4 of WWP2 by transfection of specific siRNA was efficient as revealed by Western blotting.

### WWP2 regulates Akt phosphorylation during mitosis

**Figure 5:**
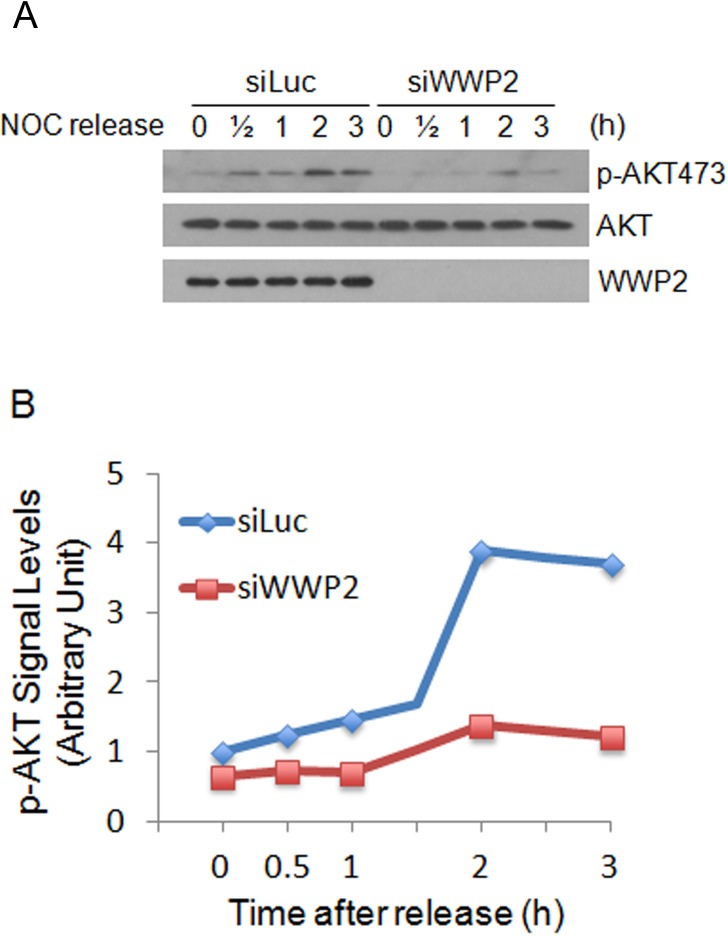
WWP2 regulates Akt activation during the cell cycle **(A)** HeLa cells transfected with WWP2 siRNAs (siWWP2) or luciferase siRNA (siLuc) for one day were treated with nocodazole for 14 h. Mitotic cells collected by shake-off were released into the cell cycle. At various times post release, cells were collected and equal amounts of cell lysates were prepared and blotted for phospho-Akt^S473^, total Akt, and WWP2. **(B)** phospho-Akt^S473^ signals in cells transfected with siWWP2 or siLuc, as shown in panel A, were quantified and the summarized data were plotted.

Akt activation is essential for cell cycle progression [12, 13]. It has been also shown that WWP2 plays a role in the positive regulation of the PI3K/Akt signaling pathway [3, 8]. To determine whether WWP2 regulates Akt activation during mitosis, cells transfected with WWP2 siRNA or luciferase siRNA were synchronized at early mitosis by nocodazole treatment and then released into the cell cycle. Cells collected at various times post release were lysed and equal amounts of cell lysates were blotted for p-Akt^S473^, an activation marker [12]. In mitotic population (time 0), p-Akt^S473^ signals were not detectable in cells transfected with WWP2 siRNA (Figure 5A). Although the signals started to increase 2 h post release the magnitude of increase was much smaller in WWP2 siRNA-transfected cells than control siRNA-transfected cells (Figure 5A and 5B). On the other hand, there was no significant change in Akt protein levels (Figure 5A). These observations strongly suggest that silencing of WWP2 significantly suppressed Akt activation during mitotic progression.

## DISCUSSION

Numerous protein components are involved in controlling cell cycle progression. Early studies have shown that the transition of cell cycle stages is driven primarily by post-translational modifications such as reversible phosphorylation [14-16]. However, other mechanisms including protein degradation also plays a crucial role in driving cell cycle progression [17-19]. Extensive studies in the past have identified and characterized two major groups of ubiquitin E3 ligases involved in polyubiquitining cell cycle regulators for proteasome degradation. They are APC/C and the SCF complexes [20, 21]. In the current study, we have identified that WWP2, a member of Nedd4-like family of ubiquitin E3 ligases, is also involved in regulating cell cycle progression. Silencing of WWP2 significantly reduces the cell proliferation rate, which is associated with the accumulation of mitotic markers including cyclin B and phospho-H3 with concomitant decrease of cyclin E.

Akt phosphorylation fluctuates during the cell cycle and its activity is required for cell cycle progression [12]. Akt activity is directly [22, 23] or indirectly [3] regulated through ubiquitin modification. As shown in Figure 2B, p-Akt^473^ signals start to rise around the mitotic entry, coinciding with the appearance and increase of cyclin B. It is possible that Akt activity may be involved in the positive regulation of expression of proteins including cyclin B that are required for mitotic entry. Double-thymidine block followed by release reveals that mitotic cells contain a unique form of WWP2 splice variant (V4). Significantly, silencing WWP2 greatly reduces phosphorylation of AktS473, strongly suggesting that it is involved in positive regulation of Akt signaling pathway. However, it is not clear how WWP2 regulates Akt phosphorylation. One possibility is that WWP2 enhances degradation of PTEN that inhibits PI3K-Akt pathway, thereby positively regulating Akt activity [3]. Further analyses are needed to elucidate how WWP2 regulates the Akt activity during the cell cycle progression. Our results suggest that the Akt activity may be indirectly regulated by WWP2 during cell cycle progression through ubiquitin modification.

We have observed that silencing of WWP2 significantly induces the accumulation of cyclin B and p-H3 in mitotic cells (Figure 4, time 0), suggesting that the absence of the enzyme arrest slows down mitotic exit. Alternatively, WWP2 may control the turnover of proteins that accelerate cyclin B turnover and dephosphorylates histone H3. On the other hand, cyclin B and p-H3 signals disappear abruptly 2 h after nocodazole release, which coincides with mitotic exit. The presence of high levels of mitotic markers coupled with their subsequent abrupt disappearance suggest that the mitotic cell population induced as the result of silencing of WWP2 may progress through the cell cycle more synchroneously and rapidly.

It is of particular interest that expression of WWP2-V4 is closely associated with mitotic entry and progression. Given its expression pattern, we propose that WWP2-V4 may play an important role in regulating mitotic components through ubiquitination. It would be of great interest to further examine whether WWP2-V4 directly targets cell cycle components, especially those involved in mitotic progression. The identification of mitotic substrates would open up new avenues of research to fully understand the function of the isoform. WWP2-V4 lacks of an N-terminal sequence that is needed for membrane association. It is logical to speculate that WWP2-V4 can translocate to the nucleus, interacting with and targeting nuclear/chromatin components. In order to understand the full function of WWP2-V4 in mitosis, it is necessary to selectively knockdown the isoform and examine mitotic progression using both cellular and molecular approaches. Alternatively, silencing of the full-length WWP2 coupled with trasnfection of WWP2-V4 isoform may also provide valuable information about the isoform in modulating mitosis.

## MATERIALS AND METHODS

### Cell Culture and transfection

HeLa cell lines obtained from the American Type Culture Collection were cultured in DMEM supplemented with 10% fetal bovine serum (FBS, Invitrogen) and antibiotics (100 μg/ml of penicillin and 50 μg/ml of streptomycin sulfate, Invitrogen) at 37°C under 5% CO2. Transfection of HeLa cells was achieved with either Fugene HD (Roche Diagnostics) following the manufacturers' protocol. Transfection efficiency was estimated to be between 80-100% in all cases through transfecting a GFP expressing plasmid (Data not shown).

### Antibodies and siRNAs

Antibodies to phospho-AKTS473, AKT, cyclin A, cyclin E, cyclin B1, α-tubulin, PARP, phospho-Histone H3 and Actin were purchased from Cell Signaling Technology. Antibody to WWP2 was purchased from Bethy laboratories. Nocodazole and thymidine were purchased from Sigma Aldrich. WWP2 siRNA (WWP2 ON-TARGET plus SMART pool) was purchased from Dharmacon. Pools of siRNAs were transfected into HeLa cells with Dharmafect I according to the protocol provided by the supplier. Briefly, cells seeded at 50% confluency in an antibiotic-free culture medium were transfected with siRNA duplexes at a final concentration of 100 nM for 24 h. Small interfering RNAs (siRNAs) targeting firefly (Photinus pyralis) luciferase (5′UUCCTACGCTGAGTACTTCGA3′, GL-3 from Dharmacon) were used as negative control for transfection.

### Cell cycle synchronization

HeLa cells were synchronized at the G1/S boundary by double-thymidine blocks. Briefly, cells were treated with 2 mM thymidine for 18 h followed by a 9 h release; these cells were treated with 2 mM thymidine for another 18 h and then released into the cell cycle for various times. For thymidine block, HeLa cells were treated with 2 mM thymidine for 18 h and then released into the cell cycle for various times. Mitotic shake-off cells were obtained from gentle tapping of either exponentially growing rounded-up cells or cells treated with nocodazole (40 ng/ml, Sigma-Aldrich) for 14 h.

### Preparation of protein extracts and immunoblotting

Total cell lysates were prepared in a buffer [50 mM Tris-HCl (pH 7.5), 150 mM NaCl, 1% IGEPAL, 0.1% SDS, and 0.5% sodium deoxycholate] supplemented with a mixture of protease and phosphatase inhibitors. Chromatin and cytosolic/soluble extracts were obtained as previously described [24]. In brief, cell extracts were prepared in the harvest buffer [10 mM HEPES (pH 8.0), 50 mM NaCl, 0.5 M sucrose, 0.1 M EDTA, 0.5% Triton X-100] containing both protease inhibitors [1 mM dithiothreitol (DTT), 2 mg/ml pepstatin, 4 mg/ml aprotinin, 100 mM PMSF] and phosphatase inhibitors [10 mM tetrasodium pyrophosphate, 100mM NaF, 17.5 mM β-glycerophosphate]. The low-speed supernatant (500g) containing cytoplasmic proteins was collected and nuclear extracts were made by vortexing the nuclei for 15 min at 4°C in a buffer containing 20 mM HEPES (pH 7.9), 400 mM NaCl, 1 mM EDTA, 1 mM EGTA, 0.1% IGEPAL CA-630, and protease inhibitors. Protein concentrations were measured using the bicinchoninic acid protein assay reagent kit (Pierce Chemical Co). Equal amounts of protein lysates from various samples were used for SDS– PAGE analysis followed by immunoblotting. Specific signals on immunoblots (polyvinylidene difluoride) were visualized using enhanced chemiluminescence (Super-Signal, Pierce Chemical Co.).

### Cell proliferation assay

HeLa cells transfected with WWP2 siRNAs or Luciferase siRNA were seeded in triplicate onto 96-well plates (2×10^3^ cells/well) and allowed to proliferate for 72 h. Cell growth was measured using a Cell Counting Kit-8 (Dojindo Molecular Technologies, Gaithersburg, MD) with the water-soluble tetrazolium salt WST-8 [2-(2-methoxy-4-nitrophenyl)-3-(4-nitrophenyl)-5-(2,4-disulfophenyl)-2H-tetrazolium, monosodium salt] as a substrate according to the manufacturer's protocol.

### Statistical analysis

Each experiment was performed at least three times. The data were plotted as the mean ± S.D. Student's *t*-test was used for all comparisons. A *P* value of less than 0.05 was considered statistically significant.
